# Can molecular stratification improve the treatment of inflammatory bowel disease?

**DOI:** 10.1016/j.phrs.2019.104442

**Published:** 2019-10

**Authors:** Claire Wang, Hannah M. Baer, Daniel R. Gaya, Robert J.B. Nibbs, Simon Milling

**Affiliations:** a Institute of Infection, Inflammation & Immunity, College of Medical, Veterinary and Life Sciences, University of Glasgow, Glasgow, UK; b Gastroenterology Unit, Glasgow Royal Infirmary, Glasgow, UK

**Keywords:** ASCA, anti-*Saccharomyces cerevisae* antibody, AMP, anti-microbial peptide, ATG16L1, autophagy-related 16-like 1 gene, CD, Crohn’s disease, CRP, C-reactive protein, EIM, extraintestinal manifestation, GI, gastrointestinal, GWAS, genome-wide association studies, IBD, inflammatory bowel disease, IFNγ, interferon-γ, IFX, infliximab, IL, interleukin, IL-10R, interleukin-10 receptor, ILC, innate lymphoid cell, mTNF, membrane-bound tumour necrosis factor-, MVKD, mevalonate kinase deficiency, NK, natural killer, NOD2, Nucleotide-binding oligomerisation domain 2, OSM, oncostatin M, OSMR, oncostatin M receptor, pANCA, perinuclear anti-neutrophil cytoplasmic antibody, RS, risk score, SNP, single nucleotide polymorphism, TGF, transforming growth factor-, Th, T helper, TNF, tumour necrosis factor-, Treg, T regulatory cell, UC, ulcerative colitis, Inflammatory bowel disease, Crohn’s disease, Ulcerative Colitis, Molecular stratification, Anti-TNF, Treatment response, Precision medicine, Disease stratification, Biologics, Faecal calprotectin, Serological markers, Genomics, Transcriptomics, Microbiomics, Proteomics, CD, UC

## Abstract

Inflammatory bowel disease (IBD) is a debilitating chronic inflammatory disease of the gastrointestinal (GI) tract. It affects more than 3.5 million people in the western world and places a huge financial burden on healthcare systems. IBD is highly heterogeneous; disease severity and outcomes in IBD are highly variable, and patients may experience episodes of relapse and remission. However, treatment often follows a step-up model whereby the patients start with anti-inflammatory agents (corticosteroids or immunosuppressants) and step-up to monoclonal anti-tumour necrosis factor-α (TNFα) antibodies and then other biologics if the initial drugs cannot control disease. Unfortunately, many patients do not respond to the costly biologics, and thus often still require gut-resective surgery, which decreases quality of life. In order to decrease rates of surgery and ineffective treatments, it is important to identify markers that accurately predict disease progression and treatment responses, to inform decisions about the best choice of therapeutics. Here we examine molecular approaches to patient stratification that aim to increase the effectiveness of treatments and potentially reduce healthcare costs. In the future, it may become possible to stratify patients based on their suitability for specific molecular-targeted therapeutic agents, and eventually use molecular stratification for personalised medicine in IBD.

## Graphical abstract

**Figure:**
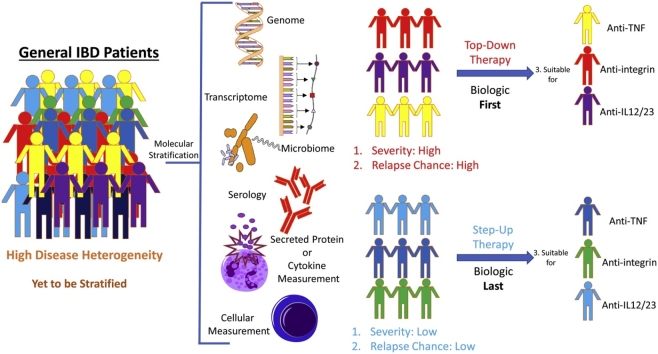

## 1 Introduction

Inflammatory bowel disease (IBD) is a term describing disease characterised by chronic gastrointestinal inflammation in a protracted relapsing and remitting course. IBD affects over 3.5 million people in the western world with incidence substantially increasing in recent decades [1]. It equally affects males and females and has a peak age of onset in the teenage years and early adulthood [2].

Crohn’s disease (CD) and ulcerative colitis (UC) are the two most common forms of IBD and differ in disease mechanism and location [3,4]. UC is a mucosal disease with continuous ulceration always affecting the mucosa of the rectum that can spread proximally into the colon [4]. A major complication of UC is the potentially life-threatening toxic megacolon, an expansion of the colon often necessitating colectomy [5]. In contrast to UC, CD can affect the entire GI tract from mouth to anus and the inflammation often penetrates the mucosa instead of solely superficially affecting the inner lining [4]. Ulcerations in CD are not continuous and occur in “skip lesions”, where healthy areas separate inflamed tissue. CD patients can experience a spectrum of complications, including strictures (narrowing of the intestinal lumen) and fistulae (abnormal connections between intestines and other organs) [6]. Unlike in CD, surgery is curative in UC [7] but it does carry significant inherent surgical risks including small bowel obstructions, sepsis, neural injury and haemorrhage [8]. Furthermore, quality of life does not return to normal after colectomy in UC [9].

IBD patients can experience a range of symptoms during active disease, including bloody diarrhoea, abdominal pain, weight loss, perianal discomfort, growth impairments, and faecal urgency to name but a few [6,10]. These symptoms have a significant impact on everyday life. As well as in increased risk of colonic cancer in patients with colonic disease [11], patients with IBD may also develop extraintestinal manifestations (EIMs). Up to 40% of patients experience pathological inflammation in non-GI organs [12], however the risk for and location of these EIMs is not predictable.

Due to the requirement of continuous monitoring and treatment to manage disease and minimise its impact on the quality of life, IBD creates a huge financial burden on healthcare systems, and has substantial indirect economic impacts [13,14]. It has been estimated that the costs of IBD in the US in 2014 was between $14.6–31.6 billion. The financial burden of IBD for the UK National Health Service in 2010 was around £3000 per patient, with overall costs of approximately £1 billion [15]. Although IBD management is expensive, only around 60–70% of patients are satisfactorily managed [16,17]. Here we discuss the limitations of current therapeutic models, the need for treatment advances in IBD and suggest the potential use of molecular stratification to improve IBD therapy. Molecular stratification distinguishes patients on a molecular level, e.g. by using genetic, transcriptomic, microbiological or proteomic information. This approach has the potential to successfully improve targeted therapy both IBD and in other inflammatory diseases such as rheumatoid arthritis.

## 2 The aetiology of IBD

IBD has a complex aetiology involving a variety of overlapping factors, including immune dysregulation, genetics and environmental factors [18]. The precise causes of IBD are unknown but, due to loss of epithelial barrier integrity in the intestine, microbes are translocated into the intestinal wall and their detection and attempted clearance by the immune system causes inflammation [19,20]. The homeostatic relationship between the intestinal immune system and commensal gut microbiota is disturbed, resulting in an abnormal intestinal immune response and subsequent pathological chronic inflammation. Immunological and genetic studies have elucidated many critical disease mechanisms.

### 2.1 Immunopathology of IBD

**Fig. 1:**
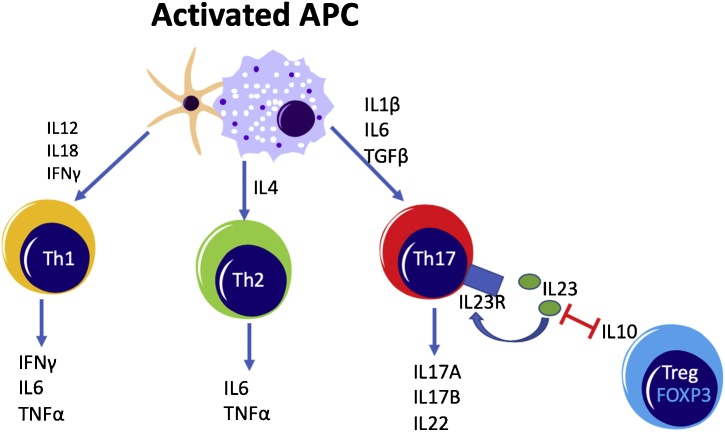
**The generation of T helper cell subsets in the intestine and their cytokine production profiles.** Th1, Th2 and Th17 cells are involved in IBD immunopathogenesis through their production of the cytokines indicated and are polarized by activated APCs. Th17 cells can inhibit FoxP3+ Treg cells through IL-23, while FoxP3+ Treg cells can suppress Th17 cells using IL-10. The Th17/Treg balance is compromised in IBD patients.

CD4 + T cells are key to the adaptive immune response and have been identified as important drivers of inflammation in IBD. Upon recognition of their cognate antigen presented by antigen presenting cells (APCs), naïve CD4 + T cells can differentiate into several distinct T helper (Th) cell types. Each expresses a unique set of cytokines and specific transcription factors (Fig. 1). Th1 cells have been described as important in the pathogenesis of CD, while Th2 cells are reported to be more involved in mediating inflammation in UC. Recent genome-wide associated studies (GWAS) have indicated that the Th17 pathway is of great importance in both CD and UC inflammation. Indeed, it is now widely accepted that a shift in the Th17/Treg balance towards pro-inflammatory Th17 cells and away from immunoregulatory Tregs is an essential component of IBD immunopathology [21,22].

To enable T cells to efficiently localise to the intestine they express specific homing receptors [23]. Intestinal endothelial cells express Mucosal Addressin Cell Adhesion Molecule-1, which adheres to the integrin α_4_β_7_ on Th and other immune cells and helps their entry into the intestine [24].. However, once the intestine becomes inflamed a large number of chemotactic receptors and cell adhesion molecules are likely to be capable of mediating T cell homing.

After localisation to the intestine T cell responses are driven by direct contact with antigen-presenting cells (APCs) but are shaped by the cytokine environment created by APCs and other immune cells. For instance, after initial activation and differentiation to Th1 (in response to IL-12) or Th2 cells (in response to IL-4), both Th1 and Th2 cells secrete tumour necrosis factor-α (TNF), a pro-inflammatory cytokine known to drive inflammation in UC and CD. In addition, upon differentiation, Th17 cells express the IL-23 receptor, enabling them to respond to the IL-23 produced in inflamed tissues, which enhances their survival [21,22]. IL-23 also inhibits Treg inhibitor, reducing their expression of the regulatory cytokine IL-10. Thus, inhibition of Tregs by IL-23 also promotes the Th17 response [21,22].

### 2.2 Genetic predisposition to IBD

From loss of epithelial barrier integrity to dysregulation of intestinal immunity, there are a large number of biological processes that contribute to the development and progression of IBD. GWAS have identified over 200 genetic susceptibility loci associated with IBD [25,26], but genetic predisposition is usually not enough for IBD onset. An exception is monogenic IBD, prevalently found in paediatric patients, where a single genetic factor causes disease [27]. The penetration of susceptibility loci is estimated to be 30% in CD and 20% in UC patients. Many of these loci are located in immune genes, including those involved in innate and adaptive immune responses, and have been associated with other autoimmune diseases. Although there are specific gene variants associated with either CD or UC, the majority are shared by both forms of IBD [25].

Although GWAS have identified many susceptibility gene variants, our understanding of how each of the identified polymorphisms contribute to IBD development is still somewhat limited. Moreover, most IBD patients are likely to have multiple low-penetrance IBD susceptibility alleles, which alone would not be sufficient to induce IBD, consistent with the multifactorial aetiology of IBD [28].

## 3 Current treatment: step-up therapy

**Fig. 2:**
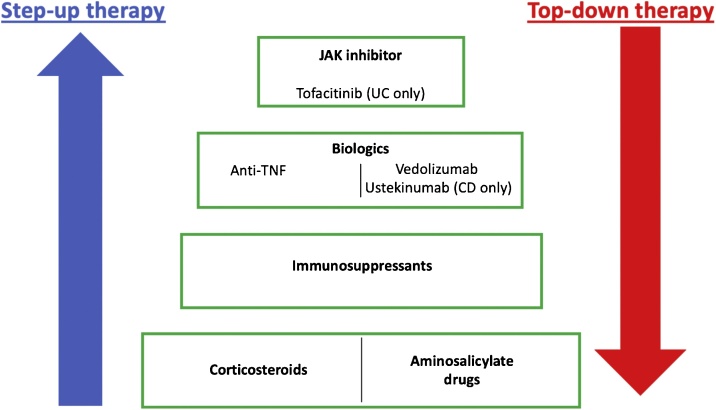
**Current approaches to IBD treatment.** Patients following step-up therapy are given frontline therapies, such as corticosteroids and aminosalicylates, followed by immunosuppressants, anti-TNF and non-TNF targeting biologics and JAK inhibitor tofacitinib (UC only) in a step-wise manner. Proceeding to the next therapy step depends on treatment responses, side effects and disease severity. An alternative treatment model, top-down therapy may be useful for patients with severe IBD. In this strategy, more specific biologics are administered sooner after diagnosis.

Despite the heterogeneity of IBD, most patients are treated using the same empirical step-up therapy approach in which increasingly powerful and generally more expensive drugs are prescribed in a step-wise manner (Fig. 2). Since there is no pharmacological cure for IBD, the step-up model focuses on controlling and alleviating intestinal inflammation by maintaining clinical remission of patients and inducing healing of ulcerated mucosa.

### 3.1 Corticosteroids, aminosalicylates and immunosuppressants

The conventional first-line treatment for many IBD patients is a corticosteroid monotherapy [29,30]. In UC aminosalicylates (5-ASA) drugs are administered as a common alternative to corticosteroids and are often sufficient to control disease, however, 5-ASA treatment is ineffective in CD [31]. Due to their severe adverse effects [32], corticosteroids are not suitable for long-term administration. Additionally, there is a wide range of contraindications that do not allow corticosteroid treatment [33]. The corticosteroid dosage is gradually lowered after clinical remission is induced, but if the patients relapse after corticosteroid withdrawal, immunosuppressants (e.g. mercaptopurine, azathioprine or methotrexate) may be are added; these can be administered long-term and used to maintain remission [29,30]. When patients are intolerant or only partially responsive to these first-line therapeutics, they may receive biologic therapy.

### 3.2 Biologics – targeted IBD therapeutics

Biologics block the action of specific molecular targets involved in IBD development and/or progression. The most commonly prescribed biologics are the anti-TNF agents, which bind the cytokine TNFα and inhibit its pro-inflammatory activity. Two commonly used anti-TNF drugs licensed for use in the EU are infliximab (IFX) and adalimumab. They have been reported to achieve up to 47% or 33% long-term remission in patients with CD (adalimumab) [34] or UC (infliximab) [35], respectively.

If anti-TNF therapy is ineffective, not tolerated or contraindicated, non-TNF targeting biologics or small molecules may be issued. Two such biologics licensed for use in the EU are vedolizumab (UC/CD) [36,37] and ustekinumab (CD only at present) [38]. vedolizumab binds α4β7 integrin to prevent homing of immune cells from the blood into the intestine. ustekinumab targets IL-12 and IL-23 by binding their shared p40 subunit (encoded by the *IL12B* gene) [38], which prevents these cytokines from binding to their respective receptors, thereby preventing the differentiation of Th1 cells and the amplification of Th17 cells. ustekinumab is currently licensed for treatment of CD, but may also have efficacy in patients with UC [39]. Very recently the Janus kinase (JAK) inhibitor tofacitinib, which inhibits enzymes involved in cytokine signalling, has been introduced into IBD for UC treatment only, as an alternative if biologic treatment failed [40,41].

The introduction of biologics has been an important advance in the treatment of IBD. Unlike corticosteroids and immunosuppressants, biologics target receptors or soluble molecules to suppress specific pro-inflammatory pathways, reducing the risks of side effects. Compared to the other IBD treatments, biologics can also induce high rates of mucosal healing, defined as the absence of ulcerations when assessed endoscopically. With front-line anti-TNF biologics, this is reported to be achieved in 44% and 46% of CD and UC patients, respectively [42,43]. High specificity gives biologics potent therapeutic benefits, however, in a heterogenous disease like IBD, high specificity increases the chance that some recipients may not respond. This might be due to the individual’s disease not being reliant on the specific protein that is being targeted.

Although the step-up therapy model described above can be effective and can allow satisfactory disease management of UC, with surgery rates dropping to 4–16% in recent years, 30–40% of CD patients still eventually require bowel surgery [16,44,45]. Increasingly, therefore, a top-down therapy model is used. Here, biologics are used to manage disease early in the treatment process for patients with severe IBD. Should remission be achieved, other treatments may be used to maintain this state. A top down strategy is increasingly employed in those presenting with severe disease at the outset and penetrating complication in CD. Such a strategy is often limited by cost and local healthcare policies.

## 4 Molecular stratification: personalised medicine for IBD treatment?

The heterogeneity and complex pathogenesis of IBD mean that a ‘one-size-fits-all’ standardised treatment, be it step-up or top-down, may not be effective. If possible, adapting the therapy to the individual characteristics of the patient’s condition would be a better treatment approach. This ‘personalised medicine’ approach aims to customise treatment according to the needs of each individual patient, based on a detailed characterisation of their disease mechanism, genetics and environmental factors. A key step towards this goal involves using molecular information to stratify patients into discrete groups.

Here, we describe two types of molecular stratification that could be integrated into the existing step-up and top-down approaches for IBD treatment. First, stratification may be used to predict disease progression (such as disease severity and risk of relapse) and treatment responses. In individuals predicted to have a milder form of disease, milder therapeutics may well be sufficient, whereas patients with a prediction for severe disease may require treatments with biologics straight away. Additionally, type of therapy or drug dosage can be adjusted sooner if a shorter clinical remission period is suspected. Second, identifying patients prior to treatment who are likely to respond to specific drugs would improve clinical outcomes, avoid unnecessary side effects, and reduce healthcare costs. With anti-TNFs costing between £3000 and £12,000 per patient annually, giving them to patients that will not respond is an expensive waste of healthcare resources.

Initially, molecular stratification could help improve the effectiveness of current treatment models by introducing elements of personalised medicine, but the ultimate aim is to move away from established treatment models and develop fully personalised medicine. Therefore, in the final part of this review, we discuss the potential of using molecular stratification as a basis for personalised medicine for IBD in the future.

## 5 Molecular stratification to predict disease progression

Predicting disease severity and outcome in IBD could inform clinical decision-making. Disease evaluation in the clinics is currently mainly based on imaging techniques and patient well-being. However, it would be very useful to introduce biomarkers that can identify inflammation before patients’ well-being is substantially affected or before they have mucosal damage detectable by imaging. This could allow prophylactic treatments to be started, and guide the decision to proceed to biologic therapy, while also helping to identify IBD patients likely to develop complications. Additionally, the dynamic nature of UC and CD makes it important to develop tools that can predict the risk of relapse. This is essential to differentiate patients that have a mild form of IBD from those that are in remission and may require adjustments in treatment to prevent relapse. Several methods are outlined here that have been employed to try to predict disease severity and outcome.

### 5.1 Serological markers

C-reactive protein (CRP) is currently the main serological marker in clinical use for IBD diagnosis and monitoring [46]. CRP is has been found to have poor sensitivity and specificity, which limits its utility. It is raised in any inflammatory illness, including infections such as flu, but is not infrequently normal in those with active IBD [47,48] Due too poor sensitivity and specificity, other serological markers are currently not in clinical use. However, peripheral biomarkers either in blood or stool samples are necessary to enable stratification, so researchers have been exploring the potential of a selection of such biomarkers.

A commonly examined serological marker in research is anti–*Saccharomyces cerevisiae* antibody (ASCA), which is often elevated in IBD patients, especially those with CD [49]. ASCA binds to mannans, a polymer of mannose in yeast cell walls. Increased ASCA in severe CD is assumed to reflect loss of immune tolerance to yeast in the gut [50]. Ferrante and colleagues studied 1538 IBD patients (75% CD; 22% UC; 3% indeterminate colitis) and found a correlation of high ASCA antibody titres with high risk of disease complications, such as fistulaes and surgery [51]. Another serological marker with potential to predict disease outcomes in UC is perinuclear anti-neutrophil cytoplasmic antibody (pANCA), which recognises proteins in the nucleus of neutrophils. An increase in pANCA suggests strong neutrophil-driven innate inflammation, which has previously been monitored in UC patients by measuring proteins associated with neutrophil extracellular traps [52]. High pANCA levels have been associated with chronic pouchitis (p = 0.03), inflammation of the ileal pouch in UC patients that have undergone colectomy [53]. In UC and CD, elevated pANCA titres have also been identified as an indicator for EIMs including uveitis (p = 0.005) and erythema nodosum (p = 0.001) [54]. An association between biomarkers and EIMs could allow additional sites of inflammation to be identified and treated more rapidly. The same study [54] also linked HLA-B27, an allele of the major histocompatibility complex I, to EIMs: 33.3% of HLA-B27-positive IBD patients in the cohort had developed ankylosing spondylitis, an inflammatory condition mainly affecting the spine and joints.

Although pANCA or ASCA have high specificity their sensitivity is low, and the tests are therefore currently not in clinical use [55]. With recent advances in understanding the disease mechanisms of IBD and the role of immune cells, other approaches could involve the analysis of cells in peripheral blood. For example, Joosse and colleagues [56] analysed the transcriptome of CD38+CD4 + T cells, which are effector T cells found at mucosal sites [56,57]. These activated T cells travel in the bloodstream before entering tissues so can be isolated from blood samples. They can express TIGIT, a receptor that can inhibit T cell activation. Based on the transcriptional profile of CD38+CD4 + T cells isolated from paediatric IBD patient blood, low expression of TIGIT by these cells correlates with shorter periods of clinical remission. Patients with <25% TIGIT + CD38+CD4 + T cells in the peripheral blood had a significantly shorter remission period than those with >25% TIGIT + cells (p < 0.05). To our knowledge, this is the first report using peripheral blood samples to monitor tissue-localised T cells and they suggest that TIGIT down-regulation may help promote T cell-driven mucosal inflammation. In order to appreciate the full predictive value of these findings, it will be important to establish the stability of these differences in TIGIT expression and understand how CD38+CD4 + T cells traffic into and out of the affected mucosal sites. This pilot study may indicate that immunopathology may be assessed in future to develop disease-monitoring markers.

### 5.2 Faecal calprotectin

Analysis of faecal calprotectin levels has revolutionised the IBD field and is used in clinical practice as first line of IBD diagnosis in addition to other diagnostic tests. A faecal calprotectin test can determine whether a patient requires further gastroenterological examinations. It is an indicator of ongoing neutrophil-driven inflammation, because it is highly abundant in the cytoplasm of neutrophils. When neutrophils rupture during intestinal inflammation, calprotectin is released into the tissue, from where it is secreted into the faeces. It is therefore a useful marker of intestinal inflammation. A pioneering study by Røseth and colleagues found, using 62 UC patients, a strong positive correlation between increased faecal calprotectin and the level of intestinal inflammation as determined by endoscopic and histologic analysis (p < 0.0001) [58]. However, this study did not identify calprotectin as a predictive marker, but rather as a marker that allows the level of active neutrophilic inflammation to be quantified at the moment of sampling.

In order to investigate its use as a predictive biomarker of inflammation, calprotectin has been studied in the context of clinical relapse. Tibble and colleagues monitored a total of 80 IBD patients with either CD or UC for one year and observed that faecal calprotectin levels of >50 mg/l had a sensitivity of 90% and specificity of 83% to predict relapse [59]. These findings were supported by a study that used 130 mg/kg of faecal calprotectin as the cut-off; the frequency of relapse was substantially higher in the calprotectin-positive group (˜59%) than the calprotectin-low/negative cohort (21%) [60]. This study also revealed that CD patients with colonic inflammation showed a significant correlation (p = 0.02) between relapse and calprotectin levels, whereas CD patients without colonic inflammation did not. In a separate study, Gisbert and colleagues studied the relationship between calprotectin and relapse in a cohort of 163 IBD patients [61]. The sensitivity (69%) and specificity (69%) to predict relapse were lower than the previous studies, potentially based on the use of a higher cut-off concentration (150 μg/g faecal calprotectin) and conjoined analysis of UC and CD. Naismith et al. have looked at the correlation between faecal calprotectin levels and relapse behaviour in CD patients over a 12 months period (n = 92). With 240 μg/ml as cut-off point, faecal calprotectin could predict relapse with a sensitivity of 80% and specificity of 74.4% [62]. A meta-analysis by Mao et al. investigated the association between relapse and faecal calprotectin levels in a cohort of 672 IBD patients (n_UC_ = 318, n_CD_ = 354) derived from 6 different studies [63]. Cut-off varied between 120 μg/g and 340 μg/g. The marker could predict relapse in IBD remission patients with 78% sensitivity and 73% specificity. Again, faecal calprotectin was reported to have a higher potential in predicting relapse in CD affecting the colon than in disease restricted to the small bowel, reinforcing the need of an additional marker for non-colonic disease.

Faecal calprotectin’s main clinical use is to distinguish IBD patients from other individuals with bowel-related conditions and to assess the effectiveness of therapies as a low calprotectin level is a surrogate maker for mucosal healing [64], but has also been established as marker for determining the risk of relapse.

### 5.3 Genomics and transcriptomics

The genome offers a huge range of potential biomarkers, some of which might prove useful for stratification. Of the >200 IBD susceptibility loci, some have already been associated with certain disease outcomes. The first gene discovered by genetic analysis to be a driver of CD was nucleotide-binding oligomerisation domain 2 (*NOD2*) [65,66]. NOD2 (also known as CARD15) is an intracellular receptor that detects peptides found on bacterial surfaces and is important for anti-bacterial immune responses. Additionally, patients with particular variants in their *NOD2* alleles have reduced production of defensin, a molecule essential for managing the gut microbiota and maintaining intestinal epithelial barrier integrity [67]. Associated with UC are polymorphisms in the *HLA-DRA* gene [68], which codes for the alpha chain of the major histocompatibility complex II, and potentially alters antigen presentation. Variation in *NOD2/CARD15* has been linked to small bowel stenosis in CD [69]; ˜80% of CD patients that are heterozygous or homozygous for this allele are reported to have developed small bowel stenosis. Bowel stenosis is a common cause of surgery in IBD [70,71] and 62% of patients requiring surgery as a consequence of this condition carried the 1007 fs variant. A larger Dutch cohort study (2804 IBD patients) [72] identified another *NOD2/CARD15* polymorphism (3020insC) that functioned as a predictor for bowel stenosis (p = 0.027) and surgery (p = 0.000172). In addition, the rs2241880 polymorphism of ATG16L1 has been associated with strictures (p = 0.005) and perianal inflammation (p = 0.035) in CD. If further studies support the prognostic value of these markers, they could help identify patients who would benefit from more aggressive immunosuppressive treatments to reduce inflammation, avoid bowel stenosis and delay surgery.

The number of interacting gene loci implicated in IBD makes it challenging to exploit the full potential of genomics to categorise IBD into discrete immunopathotypes. However, Haritunians and colleagues used multiple IBD risk loci to categorize UC patients into groups according to whether their IBD had resulted in a colectomy [73]. By analysing 929 refractory UC patients, they were able to use 46 distinct single nucleotide polymorphisms (SNPs) to define four colectomy risk groups with risk scores (RS) based on the number of risk alleles in the patients (ranging between 0–92 i.e. 46 SNPs, 2 alleles). 100% of patients in “risk group D” with the highest RS (53–60) required colectomy, whereas only 0.9% of colectomy cases were in risk group A, which had the lowest RS (28–38). This pilot study thus demonstrated a successful use of genetic information to stratify UC patients with different disease outcomes.

The use of genomics in IBD stratification opens the door to diagnose patients at a molecular level and delivers unequivocal information that is independent of when the sample is taken. This may allow risk groups to be identified, which could have clinical utility, but it fails to accommodate the highly dynamic nature of IBD or the importance of environmental factors in shaping disease progression. It is therefore highly unlikely that genomics alone will be able to robustly predict disease outcome and thus has no yet entered routine clinical practice.

By measuring gene expression, transcriptomics should be more effective than genomics at identifying different clinical states. For instance. a transcriptomic approach using blood CD8^+^ T cells from 67 patients with active IBD has been used to successfully identify two groups according to disease severity [74]. A transcriptional signature was found that was associated with severe IBD with shorter remission periods: it was based on increased expression of genes involved in IL-7 function and T cell receptor ligation. Validation in a larger patient cohort is required but, if replicated, it may indicate that the evaluation of individual cell types crucial to IBD pathogenesis could have prognostic value.

### 5.4 Analysis of the microbiome

Patients with IBD are known to have a less diverse microbiome than heathy controls [75,76]. Since a hallmark of IBD pathogenesis is dysbiosis between the mucosal immune system and gut microbiota, the microbiome has the potential to harbour species with biomarker capacity.

A recent study revealed that the analysis of the genetics of the gut microbiota could be a useful non-invasive way of localising inflammation in CD [77]. Identifying the intestinal regions that could potentially be affected by inflammation is crucial to predicting disease progression and outcome. Healthy twin pairs were compared to concordant pairs (both with CD) and discordant pairs (one healthy and one with CD). Patients with ileal or colonic CD were included. Interestingly, faecal samples from those with ileal CD were reported to have very low levels of *Faecalibacterium prasnitzii* and increased levels of *Escherichia coli* compared to healthy controls and patients with colonic CD. Acquisition of faecal samples for microbiome analysis is very simple, and processing is based on well-established techniques. However, the microbiota quickly adapt to external factors, such as diet [78], so it might be argued that they may be unreliable markers. However, this adaptability of the microbiota may allow microbial analysis to be exploited as a method to track dynamic changes in a patient’s disease state.

The potential of microbial analysis to predict relapse risk has recently been studied by Rajca and colleagues [79]. They analysed faecal samples from 33 CD patients collected at baseline, 2 and/or 6 months after first IFX treatment and then at 16 months during the follow-up period. They found lower levels of Bacteroides (p = 0.004), *Faecalibacterium prausnitzii* (p = 0.010), and *C. coccoides* (p = 0.0004) in the faecal samples of patients that experienced at least one clinical relapse during the time frame of the study. Interestingly, IFX therapy elevated the levels of these bacteria and prolonged remission. This study emphasises how using microbial analysis to assess the dynamic nature of IBD could be used to monitor and predict changes in disease over time.

Recent successes in nutritional therapies [80,81], which can reshape the microbiome composition via changes in diet, have again highlighted the importance of the microbiome in IBD pathology. The studies above are only a small selection of an emerging field with huge potential, not only in therapeutics but also disease monitoring.

## 6 Molecular stratification to predict response to biologics

Treatment with biologic agents is currently the closest therapeutics have come to personalised medicine. After assessing disease severity, IBD patients with severe disease may receive treatment with biologics. To decide which biologic is most suitable, it would be desirable to be able to distinguish likely responders from non-responders before beginning treatment. For both the step-up and top-down models, anti-TNF is the front-line biologic. Therefore, the ability to predict anti-TNF responders will improve therapeutic success.

## 6.1 Predicting anti-TNF responses

### 6.1.1 Genetic polymorphisms

Single nucleotide polymorphisms (SNPs) are potential biomarkers of clinical responses. A systematic review by Bek et al. investigated potential genetic markers for treatment response in both CD and UC, patients. 15 studies were selected with cohort sizes varying between 102–534, and treatment responses were evaluated based on clinical symptoms and/or antibody response (i.e. CRP levels) at time points between 2 and 30 weeks. SNPs associated with disease were compared to frontline anti-TNF treatment responses (IFX or adalimumab). Variants in toll-like receptor (*TLR*) *4* (rs55030728), FC fragment of IgG receptor IIIa (*FCGR3A*, rs396991), tumour necrosis factor receptor superfamily 1A (*TNFRS1A*, rs4149570), interferon-gamma (*IFNγ*, rs2439561), interleukin-6 (*IL-6*, rs10499563) and interleukin-1B (*IL-1B*, rs4848306) were related to improved treatment response to anti-TNF in IBD, whereas *TLR2* and *TLR9* SNPs rs3804099 and rs352139, respectively, were linked to a weaker response [82]. It is possible that *TNFRSF1A* rs4149570 might encode a TNF receptor more susceptible to anti-TNF treatment. SNPs in *FCGR3A* and *TLRs* can potentially be associated with different IgG antibody and antigen recognition response, respectively, however it is unclear how this impacts IFX/adalimumab efficacy. IL-6 and IL-1B are, like TNFα, products of an initial innate pro-inflammatory immune response and are released downstream of TLR signalling. Additionally, together with other cytokines, IL-6 is involved in T cell polarisation. Although similar to polymorphisms in *TLR* it is unclear how these variants affect anti-TNF treatment response.

It is known that intestinal T cells in IBD patients display dysfunctional apoptosis [83,84], and that this can be reversed by IFX treatment [85]. Hlavaty and colleagues reported a relationship between SNPs in apoptosis-associated genes (Fas ligand and caspase-6) and clinical responses to IFX treatment in CD patients [86]. In this study, 52.1% of 163 luminal CD patients reached remission 4 weeks after one dose of IFX, and patients with at least one ‘C allele’ of the Fas ligand gene were significantly more likely to recover. Interestingly, another study reported that CC genotype in Fas Ligand were four times less responsive to anti-TNF compared to TT genotype [87]. No correlations were reported between Fas ligand genotypes and any other clinical parameters, perhaps indicating that the genes influencing response to therapy might be distinct from those contributing to signs of disease.

An obvious association to anti-TNF response are polymorphisms in the *TNFα* gene. A metanalysis investigated polymorphisms in the TNFα promoter region in IBD and spondyloarthritis [88], an inflammatory disease affecting the spine and other tissues. In 352 IBD and 211 spondyloarthtitis patients two alleles, 308-*G* and 857-*C,* were identified as predictor for anti-TNF therapy responders. Although the UK National Health Service may start using genomic medicine in the near future, genome sequencing for SNP identification in IBD patients is not currently considered to bring sufficient benefit to justify the associated costs [89,90].

### 6.1.2 Immune gene expression

Due to the important role of adaptive immunity in the pathogenesis of IBD [21], it might be expected that responders and non-responders would show differences in expression of genes involved in adaptive immune responses. Toedter and colleagues performed gene expression analysis on colonic biopsies from 48 UC patients and identified a selection of genes involved with Th1, Th2, and Th17 pathways whose expression differed between those who did, and those who did not, respond to IFX [91]. Most of these genes were associated with Th17 responses and they were down-regulated in responders once they received IFX, and were lower in IFX-treated responders than IFX-treated non-responders. These gene expression patterns were validated in additional colon biopsies and could be used to distinguished responders from non-responders with an 81–100% success rate, depending on IFX dose and treatment [91]. Another study used colon biopsies from two separate UC cohorts to identify and validate differences in the expression of 5 genes between IFX non-responders and responders and show that it could distinguish between these two groups with 89% accuracy [92]. None of these 5 genes overlapped with those identified by Toedter and colleagues, but both studies generated transcriptional profiles that could be useful in predicting and monitoring responses to anti-TNF.

Verstockt et al. studied gene expression in whole peripheral blood samples of 54 IBD patients with active disease (n_CD_ = 23, n_UC_ = 30) at baseline prior to treatment initiation and followed-up treatment response for 24 weeks [93]. Reduced Triggering receptor expressed on myeloid cells1 (*TREM1*) expression was linked to anti-TNF responders in both UC (p = 0.001) and CD (p = 0.007). Analysis of the transcriptome of intestinal biopsies confirmed this target to be also downregulated in mucosal tissue of responders and additionally identified lower levels of oncostatin M (OSM), TNF and TNF receptor 2 (TNFR2). Since TREM1 could potentially function as a peripheral biomarker for anti-TNF response it is a very attractive target for future research.

Since TNF is a very important compartment of the pro-inflammatory immune response with a wide range of down-stream effects, administration of TNF blocking agents can induce changes in immune gene expression. These changes may be a contributing factor to the loss of response after initial effective treatment, another challenge the IBD field is facing. A recent study compared gene expression of IBD patients with (n = 12) and without (n = 12) anti-TNF treatment and healthy controls [94]. It was found that anti-TNF treatment impaired expression of lipocalin 2 (LCN2), an antimicrobial peptide, and Treffoil factor 1 (TFF1), an important component of intestinal mucus.

### 6.1.3 Cytokine expression

Responses to anti-TNFs may be affected by the level of TNFα in the inflamed tissue at the start of treatment. Atreya and colleagues studied the baseline level of TNF in patients by administering adalimumab conjugated to a fluorescent label to allow them to visualise membrane-bound TNF (mTNFα) in the inflamed intestinal regions using confocal laser endomicroscopy [95]. This *in vivo* imaging approach was performed on 25 CD patients who then received further therapeutic doses of adalimumab. 12 weeks post-treatment the cohort that had had relatively high numbers of mTNFα-expressing cells (mean of 30 per confocal image) had a 92% response rate, while the group with fewer mTNFα-expressing cells (mean of 11 per confocal image) had a response rate of only 15%.

Other studies have examined whether other cytokines and cytokine receptors could be useful markers of anti-TNF responses [[96], [97], [98]]. West and colleagues demonstrated that the expression of OSM can be used to predict response to anti-TNF therapy [96]. Compared to healthy controls, they detected an abundance of OSM and its receptor (OSMR) in inflamed intestinal tissue from CD and UC patients. High levels of OSM prior to IFX treatment were strongly associated with a failure to respond to the IFX. Up to 85% of patients with low baseline OSM had complete mucosal healing, whereas only 10%–15% with high levels responded to anti-TNF therapy. The authors also observed that high levels of OSM expression in UC were a predictor of patients that lost anti-TNF responsiveness ˜30 weeks after initiating treatment.

### 6.1.4 Proteomics

Improvements in the technology to quantify proteins, proteomics, have recently been led to proteomic analyses of potential IBD biomarkers. Biopsies and serum of biologic drug treatment naïve UC patients (n = 56) were analysed with quantitative proteomics to determine cytokine and anti-microbial peptide (AMPs) levels [99]. Anti-TNF responders (n = 25) showed different expression patterns of AMP or proteins associated with AMP response, when compared to non-responders (n = 31). Overall expression of defensin-5α and eosinophil cationic protein were related to anti-TNF response, whereas high cathepsin, IL-12, IL17A and TNF protein expression were indicators for non-response. In an additional anti-TNF treatment naïve UC cohort (n = 43) reduced levels of CD14 and CD86 macrophage markers and chemokine CCL2 in intestinal biopsies were linked to an improved treatment response 14 weeks after treatment initiaiton [100]. Additionally, this study detected lower cell surface expression of CD14 and CD86 on circulatory monocytes in peripheral blood samples in responders detectable 2 weeks post-treatment initiation onwards.

Gene expression analysis is useful for understanding potential disease-associated changes in gene transcription; however, transcriptomic data cannot always provide information about the functional repercussion these changes have, and does not take post-translational modifications into consideration. Proteomics and other tools of protein quantification, such as immunohistochemistry and flow cytometry, give a clearer indication about how functionality may be affected.

Overall, combining data obtained from analysing genomic polymorphisms, changes in gene expression, and changes in protein expression, provides a strong basis for understanding the complex mechanisms underlying disease response. Currently, there is insufficient evidence to support the use of biomarkers in the clinic to predict anti-TNF response [95]. However, the studies described here are important steps towards this goal; they describe potential biomarkers which, after testing in larger cohorts over longer time periods, might prove to be clinically useful in the future.

## 6.2 Predicting response to non-anti-TNF biologics

In the step-up therapy model, biologics targeting molecules other than TNF are administered to those who do not respond to anti-TNFs. It may be more useful if patients likely to respond to these drugs were identified before commencing biologics [101,102]. However, non-anti-TNF biologics have only recently been approved so studies aimed at identifying biomarkers that predict responses are in their infancy [103].

### 6.2.1 Microbiota and cellular studies in ustekinumab-treated patients

The microbiota of ˜500 anti-TNFα non-responders with moderate to severe CD was analysed prior to receiving the IL12/23-targeting drug ustekinumab [104]. When assessing responses at week 6, it was shown that ustekinumab responders had significantly higher levels of *Faecalibacterium* and *Escherichia*/*Shigella* prior to treatment than non-responders. *Faecalibacterium*, an important gut commensal, was suggested to have the most predictive value because of its ubiquity in the cohort, and they have previously been proposed as a candidate for probiotic treatment for CD [105]. However, the microbiome is highly diverse so despite the broad distribution of *Faecalibacterium* it alone might not be sufficient to predict response to ustekinumab [106].

Innate lymphoid cells (ILCs) have recently received much attention in mucosal immunology. These cells have high cytokine production capacity and can profoundly influence intestinal immune responses. In IBD, there is a bias towards ILC1s, which express transcription factors and cytokines present in Th1 cells. ILC1s are supported by IL-18, and IL-12, the target of ustekinumab [107,108]. Creyns and colleagues studied 46 CD patients that had failed treatment with anti-TNF and vedolizumab, and were due to receive ustekinumab [109]. Response was gauged by endoscopic examination 24 weeks after therapy onset. Interestingly, ustekinumab responders had significantly lower baseline levels of peripheral blood ILC1s (p = 0.017) and the authors postulated that ILC1s cells in the peripheral blood of non-responders migrate into the gut to overcome ustekinumab-mediated IL-12 blockade. Alternatively, the low levels of ILC1s in the blood of ustekinumab responders might be because more of these cells are in the gut. This could indicate that IL-12 is active in these patients and explain why they respond to ustekinumab. This is the first study to examine ILCs during biologic treatment and, if the findings are validated in a larger independent cohort, they could pave the way for using ILC1 quantitation as predictive biomarker of ustekinumab response [109].

### 6.2.2 Expression studies to predict response to vedolizumab

Boden and colleagues studied peripheral blood from 15 CD and 11 UC or unclassified IBD that were refractory to anti-TNF and due to receive vedolizumab, the α_4_β_7_ blocking antibody. Interestingly, α_4_β_7_ expression on CD4^+^ T cells and Natural Killer (NK) cells prior to treatment was found to be higher in responders than non-responders [110]. A separate study of 11 CD and 17 UC patients found that vedolizumab non-responders had higher baseline levels of circulating IL-6 [97], and that CD40 Ligand (100% specificity/sensitivity) and osteocalcin (100% specificity, 85% sensitivity) could predict response in CD or UC patients, respectively. These observations are encouraging, but the cohort size was small in both of these studies and further validation is required.

The studies discussed above show that approaches are emerging that could be used to stratify patients based on their likely responsiveness to biologics [95,96,104,105,110]. There are many factors that will determine the form of IBD a patient has and their response to biologics, including genetic predisposition, physiological condition, disease severity, the microbiome and immune function [111]. Gene expression and cell phenotypes are likely influenced by all these factors so perhaps these might be the most suitable was of predicting therapeutic responses [112]. To reduce patient discomfort, and maximise utility, it is desirable to assess these in easily accessible material, such as peripheral blood. The studies described above provide reasons for optimism but they need to be validated in larger patient cohorts before they enter the clinic. We can expect more progress in the near future.

## 7 Molecular stratification for personalised medicine

**Fig. 3:**
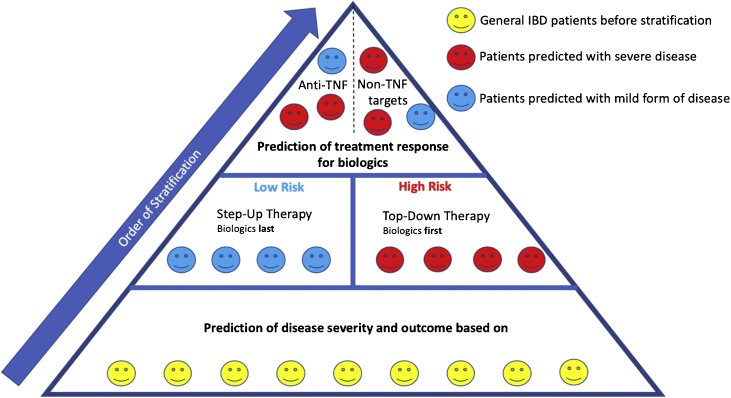
**Molecular stratification of patients with IBD.** Patients following conventional therapy might be stratified based on predicted disease severity and outcome to determine if aggressive or milder therapeutics should initially be given. Subsequently, the patients may be further stratified based on their likelihood of responding to specific biologics.

The stratification approaches described above could be used to inform progression through the step-up and top-down IBD treatment models. However, the ultimate aim is to move away from these models and use personalised medicine to direct treatment (Fig. 3). In this section, we discuss whether molecular stratification can be used to identify the unique disease drivers in each patient, and explore the possibility of more broadly providing personalised medicine for IBD patients.

### 7.1 Monogenic IBD

The majority of IBD patients have polygenic disease, but in some paediatric patients with early onset IBD disease is driven by high penetrance alleles or caused by the dysfunction of a single gene [106,113]. The first genes associated with monogenic IBD were those encoding IL10 and its receptor (IL-10R). Kotlarz and colleagues identified ‘loss-of-function’ mutations in either *IL10* or *IL10R* in ˜25% of patients with severe infantile IBD (refractory colitis and perianal disease within the first 3 months of life) [114]. Five patients with *IL10R* mutations received stem cell therapy to restore IL-10R-mediated signalling, and this induced clinical remission in all patients for at least 2 years [114].

Other monogenic causes of disease have also been identified. In these rare conditions, specific molecular therapies can sometimes be developed. For instance, a ‘gain-of function’ mutation in the NLR family CARD domain-containing protein 4 (*NLRC4*) gene was identified in a 9-week-old patient who developed early-onset enterocolitis and had unusually elevated levels of serum IL-18 [115]. NLRC4 activates the inflammasome, a molecular complex required to induce pro-inflammatory cytokines, such as IL-18. Recombinant human IL-18 binding protein was prescribed to inhibit IL-18: it reduced symptoms of enterocolitis and caused an acute drop in serum inflammatory markers [113, [115].

Thus, molecular diagnoses and personalised medicine can be used to successfully treat some IBD patients. However, monogenic IBD accounts for only a small percentage of IBD [113,116], and applying personalised medicine to polygenic IBD is far more challenging, where genetic information alone is unlikely to be sufficient [101,113,117].

### 7.2 Molecular stratification of polygenic IBD

In order to develop personalised treatment in polygenic IBD, studies are currently attempting to elucidate molecular disease mechanisms of IBD in more detail. There are, as yet, no examples of successful personalised therapy in polygenic IBD, i.e. the use of patient information to develop a tailored therapy. However, given the volume of studies examining the molecular pathology of IBD and the recent rapid progress in this field, we are optimistic that such examples will soon begin to appear.

## 8 Conclusions

Rapid progress is being made in molecular stratification for IBD, which is now beginning to allow prediction of disease severity and the risk of developing complications. Currently the best potential stratification strategies utilise serological and faecal markers. Few transcriptomic and metabolomic studies have shown sufficiently promising stratification results, but with further research they can be expected to produce more effective ways to stratify in the future.

Development of new forms of stratification will not soon replace the current methods of diagnosis (e.g., endoscopy), but will likely facilitate non-invasive disease monitoring and potentially enable more effective choice of the best treatments. Thus, if a patient is predicted to have severe disease together with short remission periods, more aggressive biologics (top-down therapy) and more frequent monitoring would be recommended to prevent surgery and complications. Patients predicted to have milder disease could potentially follow the step-up therapy model.

Biologics targeting TNF, α4β7 or IL-12/IL-23 are effective for many, but not all patients. Since not every patient responds to these drugs, molecular stratification is desirable to predict the likelihood of response. Candidate biomarkers to predict therapeutic response include molecules involved in Th1, Th2 and Th17 pathways. The expression of *oncostatin* M and mTNFα in the gut, for instance, were reported to be reliable for differentiating between responders and non-responders prior to anti-TNF treatment. In addition, studies of stool samples identified microbial biomarkers to predict the response towards ustekinumab (anti-IL-12/IL-23). Furthermore, measurements of the level of α4β7 integrin expressing cells in peripheral blood can predict responses to the anti-α4β7 biologic vedolizumab. Unfortunately, none of these biomarkers are yet proven to be suitable for use in routine clinical practice.

Despite progress in the field there are still not biomarkers available that can be used in the clinic for prognosis or to predict treatment response, and the clinical need for such biomarkers is high. Ideally, IBD patients would receive tailor-made treatments upon diagnosis. Although this may currently be possible for rare cases of monogenic IBD, more studies elucidating the immune pathways contributing to polygenic IBD are needed before such personalised medicine can be generally applicable. Although we are still far away from truly personalised medicine in IBD, molecular stratification is already beginning to be used. We are optimistic that by expanding the use of molecular analyses beyond serological markers, both clinical and cost effectiveness of IBD therapy can be further enhanced in the future.

## Conflict of interest statement

**S Milling** received speaker’s fees from Janssen and participated in medical board meetings with Pfizer. He also receives fees in his role as Editor in Chief of *Immunology*. The rest of the authors have no conflicts of interest to disclose.
